# Cost and effectiveness of water, sanitation and hygiene promotion intervention in Ghana: the case of four communities in the Brong Ahafo region

**DOI:** 10.1016/j.heliyon.2018.e00841

**Published:** 2018-10-04

**Authors:** Paa Kwesi Woode, Bismark Dwumfour-Asare, Kwabena Biritwum Nyarko, Eugene Appiah-Effah

**Affiliations:** a Civil Engineering Department, Kwame Nkrumah University of Science and Technology, Kumasi, Ghana; b Environmental Health & Sanitation Department, Faculty of Science & Environment Education, University of Education, Winneba, Asante-Mampong Campus, Ghana

**Keywords:** Public health

## Abstract

Knowledge of cost and effectiveness of Ghana's main hygiene promotion intervention (HPI), Community-Led Total Sanitation (CLTS), is critical for policy direction. Cost and resultant effect of HPI is examined using a case study of four communities. Surveys were conducted with 300 households, CLTS implementers and relevant agencies during the study period (May 2012 to February 2014). The HPI produced marginal but statistically significant effect (8%, p < 0.001). Improvement in hygiene behaviour was statistically associated with both government investments (p < 0.001) and household investments (p < 0.001). Actual HPI cost is US$ 90 per household: US$ 51 and 39 from government and households respectively. Cost-effectiveness of the HPI is US$ 106.42 per capita of improved hygiene behaviour.

## 1 Introduction

Hygiene practices are invaluable in improving public health (Kemeny, 2007; Pruss-Ustun et al., 2014). Pruss-Ustun et al. (2014) estimate that in 2012 over 1 million deaths were attributable to inadequate and poor sanitation, drinking water and hand hygiene. There is significant incremental effect of hygiene intervention to water and sanitation (Mahon and Fernandes, 2010; Mason et al., 2013; Lawan et al., 2010). For emphasis, it is asserted that supply-led sanitation provision in the form of infrastructure subsidies or direct investment may not be effective at reducing diarrheal diseases risk if good hygiene behaviours are not promoted (Potter et al., 2011; Rheinlander et al., 2010).

Upon realization of the critical role hygiene plays in achieving significant positive impact from water and sanitation interventions, several approaches have arisen to promote hygiene as an integral part of any WASH (water, sanitation and hygiene) intervention. Community-Led Total Sanitation (CLTS) is an example of such hygiene promotion approaches that is aimed at changing behaviours positively towards sanitation and hygiene (Kar and Chambers, 2008). In practice, CLTS is a participatory community-centred approach to improving sanitation coverage by increasing hygiene awareness through the use of shame (Kar and Chambers, 2008). CLTS aims to raise community consciousness and to stimulate improved hygiene behaviour and sanitation practices (Kar and Milward, 2011; Chambers, 2009).

In Ghana, CLTS has been adopted with supporting policy framework as the main sanitation strategy since 2011 (Ampadu-Boakye et al., 2011). There is however, at present, limited evidence on the resultant effects and also the associated costs of this sanitation and hygiene intervention and/or its variants (Sijbesma and Trea, 2009). This study was conducted as part of preliminary efforts to investigate the evidence of cost effectiveness of CLTS interventions in Ghana. Investigating cost effectiveness of CLTS interventions is critical for improvement of the CLTS processes within the Ghanaian context as well as providing proof of the usefulness of the approach to policy makers, development partners and all other stakeholders (Van De Reep, 2010).

## 2 Study area

**Fig. 1:**
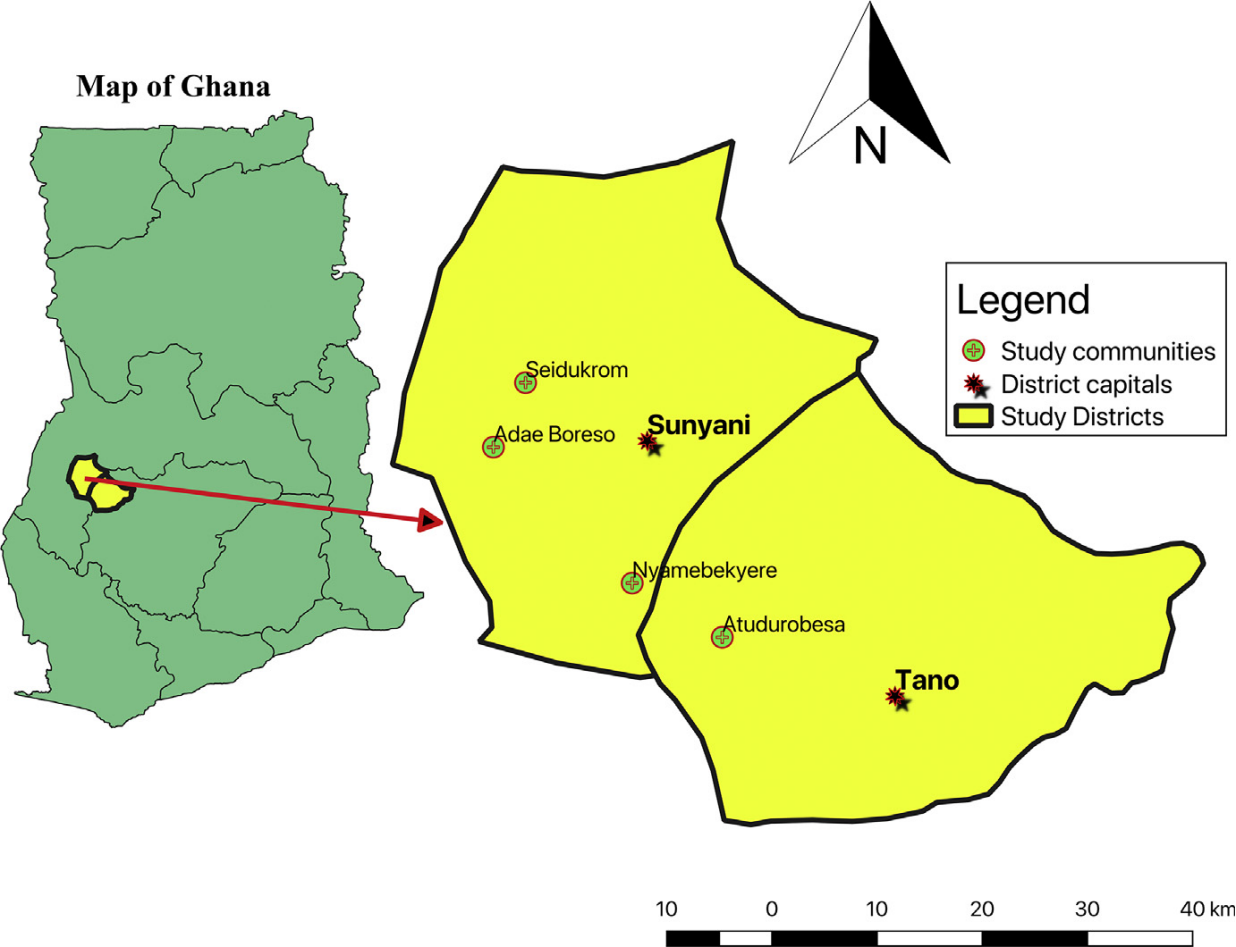
A map showing the location of the study communities in their respective districts of Ghana.

The study was undertaken in four communities namely Seidukrom, Atudurobesa, Nyamebekyere and Adae Boreso. The communities are situated within the Tano North Districts and Sunyani Municipal Assemblies of the Brong Ahafo region of Ghana (Fig. 1). The communities have small populations ranging between 300 and 600 with the number of households between 60 and 120. The Hygiene Promotion Intervention (HPI) under study in the communities was part of a larger project called Peri Urban Rural and Small Town Water Supply and Sanitation Project (RSTWSSP) in the Brong Ahafo region of Ghana. The specific HPI was Community-Led Total Sanitation (CLTS) that was aimed at improving health outcomes through improved hygiene and sanitation in the communities. A local non-governmental organisation (LNGO) was contracted to carry out the CLTS intervention which involved carrying out CLTS triggering, follow-ups, and supervising the formation of Water and Sanitation Management Teams (WSMTs) within HPI communities. The CLTS intervention was complemented with subsidy-based provision of access to safe water supply (boreholes fitted with hand-pumps) to beneficiary communities.

The CLTS interventions and the larger RSTWSSP project were implemented by Community Water and Sanitation Agency (CWSA), which is the main government agency under the then Ministry of Water Resources, Works and Housing (MWRWH) responsible for the facilitation of rural water and sanitation services delivery in Ghana. Thus, until the creation of the new Ministry of Water Resources and Sanitation under a new government, CWSA operated under the MWRWH. The CWSA also coordinated the HPI through its regional program efforts, and provided institutional support and post-intervention monitoring. The District Water and Sanitation Teams (DWSTs) of the local Assemblies under the Ministry of Local Government and Rural Development (MLGRD) provided support to the CWSA in terms of selecting and locating HPI beneficiary communities, and also during monitoring and evaluations of the HPI.

## 3 Methodology

The research design was an intervention study which compared the effects of treatment on a subject with the status of the same subject before treatment (Hennekens and Buring, 1987; Kleinbaum et al., 2007), and hence did not use matching controls for study communities. All households (estimated at 376 with an average of 94 households per community) in the four study communities were targeted for questionnaire surveys. However, the turnout or success rate of households responding or participating in the surveys was around 68% (N = 257). Households which were not captured (32%) either intentionally exempted themselves and/or were not present at the time of the surveys. The household surveys used structured questionnaire and observations to capture various data including costs, hygiene behaviour, and household sanitation practices. The household surveys were conducted in three rounds or at three time points: 1) before the commencement of the HPI, which is the baseline survey in 2012; 2) just after the HPI communities were declared open defecation free (ODF) status, which is the midline survey in 2013; and 3) finally one year after achieving ODF, which is the endline survey in 2014.

Data on cost of HPI implementation were also collected from relevant stakeholders including government agencies and a local non-governmental organization (LNGO) that was identified as the implementing partner. Informed consent for approval of the study was sought from the local authorities (Assemblies) and traditional leaders in the communities. All household respondents willingly participated in the study after giving their informed consent.

## 4 Analyses

The data analyses involved two levels: household hygiene status analysis, and the hygiene cost and effectiveness analyses. Household hygiene status data was analysed based on hygiene assessment framework adapted from Potter et al. (2011) and Dubé et al. (2012) (Table 1), which assesses household hygiene behaviour based on three key indicators: 1) faecal containment and latrine use, 2) hand washing with soap, and 3) drinking water source and storage management. The levels of the indicators are simplified into two basic practice levels, which are defined as either adequate or inadequate (Table 1).

**Table 1:** Household hygiene behaviour assessment framework.

Hygiene practice level	Hygiene assessment indicators defined		
	Faecal containment and latrine use	Hand washing with soap	Drinking water source and safe storage
Adequate	Household uses latrine that separates users from faeces (or practice dig and bury when latrine is not available) including safe handling of infant/new born faeces	Household washes hands with soap during at least two critical times: before eating and after defecation. A dedicated facility is/must be present	Household always fetches water from safe sources and safely covers stored water
Inadequate	Household latrine does not separate users from faeces, or household defecates in the open	Household does not wash hands with soap at any of the two critical times, after defecation or before eating.	Household does not always use water from safe sources

The basic assessment framework was adapted by making few modifications. Our modifications were: inclusion of infant and new born faecal management (thus making it more explicit as part of the principle of all inclusive household faecal containment); restricting ourselves to hand washing with soap only but not soap substitutes; and finally defining an overall hygiene behaviour indicator by combining all the three key hygiene behaviour indicators. Infant and new born faecal management (now explicit in our framework) is key in the reduction of incidence of diarrheal disease amongst infants (Godana and Mengiste, 2013; Islam et al., 2018). Although soap substitutes could be as good as soap itself (Potter et al., 2011), however, they stand the risk of becoming contaminated and being disease reservoirs themselves (Bloomfield and Kumar, 2009), hence soap substitutes are not regarded as adequate in this study. Aggregation of indicators to give an overall hygiene level as adequate or inadequate provides a single hygiene level measure to allow matching against HPI cost. For instance, the overall hygiene behaviour (adequate or inadequate) is the final or resultant hygiene status of a household after combining the levels of all three hygiene behaviour indicators. The preference is given to adequate hygiene level, which stipulates that at least any one of the two critical indicators (faecal containment and latrine use; and handwashing with soap at critical times) must be met. Although all three indicators are relevant but emphasis is placed on the two indicators which are considered critical based on the knowledge of diarrheal diseases and hygiene behaviour nexus (Iyer and Sara, 2005; Zwane and Kremer, 2007; Talaat et al., 2011; Freeman et al., 2017; Islam et al., 2018). Already, reviews strongly suggest that the first two indicators (faecal containment and latrine use, and hand washing with soap at critical times) can independently control diarrhoeal disease risks, and the third indicator “drinking water source and safe storage” provides significant additional benefit when any one or both former indicators are effective (Waddington and Snilstveit, 2009; Freeman et al., 2014; Dreibelbis et al., 2014). This does not discount the significant effect some studies have found for household water treatment at point of use (Arnold and Colford, 2007; Waddington and Snilstveit, 2009), which was not promoted under the HPI in our study.

The HPI costs are disaggregated into the WASH cost components of capital expenditure (CapEx), operational expenditure (OpEx), and expenditure on direct support (ExpDS). Thus, CapEx is one time investment cost of programme implementation and/or acquisition of fixed assets; OpEx is operations and minor maintenance cost; and ExpDS refers to the back-up support to keeping services running including monitoring, training and technical support to communities and service providers (Burr et al., 2012; Smits et al., 2011; Reddy and Batchelor, 2012). The OpEx and ExpDS are part of recurrent cost, which is the routine expenditure expected to sustain service over time (Smits et al., 2011; WASHCost Project, 2012; Dwumfour-Asare et al., 2014). For the hygiene cost and cost effectiveness analysis, the costs are defined in details in Table 2. Apart from financial costs which are captured as reported or by records available, cost for time in man-days spent on HPI related activities such as latrine construction and attending intervention meeting sessions (Table 2) was valued based on reported average daily wage of US$ 4 (around GHS 10) per person. The cost of the three key hygiene assessment indicators was determined from cost of activities associated with the respective indicators (Table 2). The aggregate cost for each indicator is referred to as indictor's cost or cost of indictor components contribution to the respective indicators (Table 2). Indicator cost is only used to find cost effectiveness for specific hygiene indicator. Certain cost components are repeated, for instance, general HPI activities cost which also contribute to attaining these indicators apart from other major HPI targets. This approach is adopted because there is no clue of the proportion of HPI effort that could be attributed to our specific indicators. However, the indicators' costs were not double counted in the total HPI cost. This is also because the total HPI cost was not derived from the individual indicators' costs. The cost values were adjusted to their equivalent values in US$ for the 2013 year by using Gross Domestic Product (GDP) inflation and currency conversion factors from the World Databank (World Bank, 2014a). This cost adjustment to current values was necessary in order to account for changes in the currency value over time (Dwumfour-Asare et al., 2014; Ristinmaa et al., 2013).

**Table 2:** Hygiene behaviour indicator cost components defined.

Cost components	Indicator 1: Faecal containment and latrine use	Indicator 2: Handwashing with soap	Indicator 3: Drinking water management
Costs of household participation in attending intervention	✓	✓	✓
Costs of community latrine building	✓		
Households expenditure on soap		✓	
Expenditure on NGO implementer	✓	✓	✓
Government Expenditure on monitoring	✓	✓	✓
Costs of water supply (hand pump + borehole)		✓	✓

Cost effectiveness was determined by dividing the cost of intervention (HPI) by the number of persons with positive change in hygiene behaviour. The approach used is based on the principle of cost per unit effect (Sijbesma and Trea, 2009), where the unit of effect is the number of persons with improved hygiene behaviour as a result of the HPI. The binary logistic form of generalized estimating equations were used to assess any association (Harrell, 2015) between cost funding sources and levels of improvement with all the indicators developed for the intervention. Binary logistic regression was employed because the response variable, hygiene behaviour, was dichotomous while independent variable of cost varied across household. Generalized estimating equations adjusted for similarity within communities. All data processing and analysis were done using Microsoft Excel, Stata 12™ and IBM SPSS packages.

The study also recognises the following as limitations – small sample sizes for study communities and households; short term assessment of the HPI cost effectiveness may not be entirely fair because HPI effect on hygiene behaviour change may not be in the short and immediate terms; limited data for detailed socioeconomic profiling of responding households; and inability to disaggregate cost of soap for handwashing from other domestic uses. Conclusions are therefore drawn bearing in mind the small sample size with limited scope of study, and other limitations. The cost effectiveness analysis (cost per capita change) is only limited to immediate gains and not the long-term effect, which may or may not yield potential balance between cost and creditable achievements over a significant time period.

## 5 Results and discussion

### 5.1 Brief socioeconomic profile

The households interviewed had an average household size 5 ± 3.2 (SD, standard deviation) persons and a total population of 1,315 (for all households interviewed) representing 67% of the total population of the communities (1,960). The monthly income, from 218 households who were able to share such information, was in a wide range of US$ 3.4–134.6 making the median US$ 42.5 (GHS 100) a better estimator of central tendency rather than the mean US$ 45 ± 27.9 (GHS 105.8 ± 65.6). The proportion of poor households identified among these households was very high (96%, i.e. 209 out of 218) and this is based on the comparison of household per capita income with the World Bank global poverty line of US$ 1.25 per capita/day (World Bank, 2014b). This observation could be confirming the assertion that incidence of poverty is very high in rural Ghana (Adjasi and Osei, 2007; Cooke et al., 2016). Majority of households (55%, N = 257) had children under five years old and this validates inclusion of infants' and newborn babies' faecal management consideration in household hygiene behaviour. The number of children under five years old from the interviewed households was around 19% (n = 372) of the total population of respondent households (1,315). Male-headed households were dominant among study households (86%, n = 221), and this figure is far higher than the national coverage of 70% (GSS, 2014).

### 5.2 Hygiene status of households

The study shows that before the implementation of Hygiene Promotion Intervention (HPI), only few households (3%, N = 257) had adequate hygiene behaviour practices which was attributed to hand washing with soap, and safe drinking water source and storage. It is also revealed that there was improvement in the proportion of households that had adequate levels of overall hygiene behaviour following the project intervention from baseline to midline (+4%), and through to endline (+12%) (Table 3). Thus, cumulatively the HPI by the time of endline improved the hygiene behaviour practices of about 31 households (12%, N = 257) and this number translates into 155 people based on the average household size of 5.

**Table 3:** Improvement in hygiene behaviour levels (HBL) across study period.

Hygiene indicators	% Households with adequate HSL at Baseline	% Improvement in HSL		
		Baseline to Midline	Midline to Endline	Baseline to Endline
Overall HSL	3	+4	+8	+12
Faecal containment & latrine use	0	+1	+2	+3
Hand washing with soap	3	+4	+6	+10
Drinking water source & storage	34	+18	−7	+11

Statistical test revealed that such improvement in proportions of households with adequate hygiene behaviour between baseline and endline was significant (p < 0.001). This suggests that the intervention made some significant immediate impact on households' hygiene behaviour within the short period. However, the different indicators had varying levels of improvement and the least improvement at all times is associated with faecal containment and latrine use (Table 3). This could be partly attributed to the slow latrine construction uptake which is normally associated with CLTS interventions and more so because most of the time emphasis is placed on achieving open defecation free (ODF) status first before moving up the sanitation ladder (Sigler et al., 2014).

Looking at individual indicators, safe drinking water source and storage had strong immediate improvement after the intervention, even though household practices at the baseline was already high (Table 3). However, there was serious retrogression between midline and endline for this same indicator. This observation could be attributed to the breakdown of improved water sources in two of the study communities, affecting approximately 190 households, and also 18% reduction in number of households that safely covered their stored drinking water (a drop from 67% at midline to 49% at endline). The observation partly underscores the point that water infrastructure or facilities are important drivers for improved hygiene practices (Curtis et al., 2000; Papafilippou et al., 2011). Also slippage in adopted hygiene behaviour is observed when fatigued and/or the stimuli of motivation declines or is withdrawn like in the case of refusing to cover stored drinking water after sometime. Hand washing with soap behaviour also saw a steady improvement due to the HPI from midline to the endline (+4% to +10%).

For faecal containment and latrine use, improvements were marginal because only 3–8 households (1–3%, N = 257) attained adequate service level. Meanwhile, interactions with study participants suggested that participants were motivated to reduce incidence of open defecation (OD). The slow improvement was hinted to be partly due to financial and technical challenges that are the main setbacks for rapid uptake of household latrine construction. This opinion was expressed during interviews with Water and Sanitation Management Teams (WSMTs) within all four communities. Other studies have recognized similar challenges, especially in situations where latrine sharing is not a taboo and income levels are also low (Rheingans et al., 2006; Movik, 2008; Thys et al., 2015). It is possible that continued investment in hygiene intervention activities, post intervention monitoring and technical support could increase and sustain improved positive hygiene outcomes, including marginal achievements (Movik, 2008; Papafilippou et al., 2011).

### 5.3 Costs of the hygiene promotion intervention (HPI)

Table 4 shows the costs of the entire hygiene intervention over the study period. The overall hygiene intervention cost, from all household and government investments (financial and non-financial like time cost), was estimated as US$ 68,615. This cost translates into US$ 37 per capita (i.e. US$ 183 per household). The initial cost of the HPI between the baseline and midline is more than twice that for the midline to endline period (Table 4). This was expected because fewer intervention activities happened between midline and endline. The government funded activities in this period (midline to endline) were mainly monitoring and evaluation, supervisions and backstopping. The baseline to midline period, in contrast, focused on the main and several HPI activities including toilet construction, water facilities provision, sensitization and awareness creation, hygiene promotion, community gathering and meetings, government agencies visits and monitoring.

**Table 4:** Costs of hygiene promotion intervention over the study period.

Hygiene indicators	Intervention period and cost (US$) Total cost (per capita cost)	
	Baseline to Midline	Midline to Endline
Faecal containment and latrine use	6,304 (3)	246 (0∗)
Hand washing with soap	35,802 (19)	21,054 (11.3)
Drinking water source and storage	21,541 (12)	246 (0∗)
Overall HPI∗∗	47,561 (25.4)	21,054 (11.3)

∗ Costs per person too small (negligible) to be represented; US$ 1 = GHS 2.35 (2013).

∗∗ The overall HPI cost is not the sum of individual indicators but the actual total expenditure on HPI explained in text.

Associating the cost with indicators showed that cost related to handwashing with soap was relatively high, followed by drinking water source and storage (Table 4). The handwashing cost was mainly expenses on soap for all uses, including handwashing, according to respondents. The respondents could not disaggregate expenditure on soap to isolate cost solely associated with hand washing. However, participants asserted that the HPI increased sensitization on general soap usage including handwashing with soap. The cost related to faecal containment and latrine use was least because very few households were able to constructed latrines, and/or otherwise regularly used shared latrines.

Disaggregation of the HPI cost into WASH cost categories showed that the least cost contribution came from Expenditure on direct support (ExpDS) - (1%), then Capital expenditure (CapEx) of entire intervention both hardware and software – 48%, and the highest from households' operational expenditure (OpEx) – 51%. While the OpEx from households is solely expenditure on soap borne by households in relation to handwashing and hygienic cleansing practices, CapEx is borne by both the government (56%, covering water supply facilities provision, and contract fees for LNGO to implement HPI), and households (44%, covering cost of time for participation in HPI meeting sessions, and time cost for constructing new communal latrines). In addition, the government incurred ExpDS covering the cost of monitoring and evaluation, technical support, and backstopping provided by staff of CWSA and DWSTs. Conventionally, ExpDS is a kind of recurrent cost that could be considered as OpEx to the government agencies CWSA and DWST for their routine task of monitoring and supervision of hygiene behaviour and practices in communities. This also implied that beyond the HPI implementation, the supporting government agencies (CWSA & DWST) would still need a routine expenditure of at least 1% of HPI cost as ExpDS, equivalent to US$ 1.8 per household. This recurrent cost may be imperative to support post intervention activities and for continuous improvement and sustainable positive change in hygiene behaviour.

Study participants believed that their expenditure on soap increased because of their increased awareness of handwashing with soap from the HPI intervention. Removing household OpEx (solely the cost of soap that accounts for 72%) from the cost of HPI will allow for comparison with one of the most recent HPI cost studies in Ghana (Crocker et al., 2017). With household OpEx excluded and focusing on only investment cost, total HPI cost considered as the typical HPI investment was around US$ 90 per household. By this HPI cost, the government paid around 57% (i.e. US$ 51) while households paid 43% (US$ 39) as their respective investment commitments to the intervention. Without OpEx, the HPI cost of US$ 90 per household in our study was within the cost range US$ 38.27–103.92 per household we deduced from Crocker et al. (2017) for a similar HPI implementation in Ghana. Again our present study showed that the government cost (i.e. HPI programme cost) of US$ 51 per household was also within the cost range US$ 30.34–81.56 per household established by Crocker et al. (2017). However, the cost to households (local investment) in this study is higher than the figures US$ 7.93–22.36 per household found by Crocker et al. (2017). This difference could be due to higher or wider coverage (close to 6,800 households) in that study (Crocker et al., 2017) and therefore the large number of recipients most likely helped to reduce their observed unit cost by economies of scale.

### 5.4 Cost effectiveness of hygiene promotion intervention (HPI)

The HPI was an investment to trigger improved hygiene behaviour practices in the beneficiary communities and therefore government's part of the HPI cost was critical since that stimulated local investment from households. The analysis of government's cost incurred to cause any change in improvement of hygiene behaviour is presented in Table 5. The analysis is presented based on the understanding that a unit percent (1%) of improvement in behaviour change is equivalent to 3 households (i.e. 15 people). The cost was highest from the baseline to midline since all major activities were undertaken within that period. Meanwhile, by the end of the intervention (from baseline to endline), the HPI had spent US$ 106.42 for a per capita conversion of safe hygiene behaviour. The government spent that amount mainly on CLTS implementation, provision of water supply systems to support effective HPI, and monitoring and supervision (by government agencies). The cost appears to be high probably because of the measurement of the HPI's immediate effect rather than medium- and/or long-term impact assessment over longer period of time after implementation of the intervention. It is also important to note that diminishing government investment per capita from midline to endline (US$ 2.04 per capita) did not reduce uptake of positive hygiene behaviour. This is probably because minor activities like monitoring were still going on and also community members still had lingering memories of awareness created during the sensitization phase of the HPI.

**Table 5:** Government costs of hygiene service level (HSL) per capita improvement.

Period of assessment	Persons with improved HSL	Total costs (US$ 2013)	Cost per Capita improvement change in HSL (US$ 2013)
Baseline to midline	60 (4%)	18,910.27	315.17
Midline to endline	120 (8%)	245.93	2.04
Baseline to endline	180 (12%)	19,156.20	106.42

Analysis for association between HPI investment sources and level of hygiene behaviour improvement at household level, using generalized estimating equations (GEE) is presented in Table 6. Improvement in the overall hygiene behaviour level is strongly associated with government's investment (p < 0.001) likewise households' or local investment (p < 0.001). This suggests that even though government expenditure led change in hygiene levels, but without a corresponding household expenditure, there would be no significant positive effect in the resultant hygiene behaviour. The same claim may be applicable to improvement in hygiene indicators for faecal containment and handwashing with soap.

**Table 6:** Association between cost funding sources and hygiene service level improvement.

Hygiene indicators	Funding sources	Significance
Overall hygiene behaviour	Government	<0.001
	Household	<0.001
Drinking water source and storage	Government	0.458
	Household	0.458
Faecal containment and latrine use	Government	<0.001
	Household	<0.001
Handwashing with soap	Government	<0.001
	Household	<0.001

Drinking water management and storage indicator levels were not significantly linked to either government or household expenditure (p = 0.458). This could be the situation probably because the intervention did not effectively target household water storage and management, and also expenditure at the household level did not include improving household drinking water safety planning and management.

## 6 Conclusions

The Hygiene Promotion Intervention (HPI) and in this case Community-Led Total Sanitation (CLTS) made significant immediate impact towards improving hygiene behaviour among some households at the end of implementation. The effect of uptake of toilet construction was very low immediately after the intervention, probably because enough time was needed to translate the effect of HPI impact on toilet construction. Meanwhile, the HPI investment cost was around US$ 90 per household where the government (HPI programme) paid for the majority (57%, US$ 51 per household) and the households paid US$ 39 per household (43%). The cost effectiveness of the intervention by the short-term assessment (immediately after intervention) was US$ 107 per capita improvement in hygiene behaviour. Both government (HPI programme) and household investment contributions were significant to achieve any unit of improved effect in hygiene behaviour. In as much as government investments in HPI are critical to trigger change in hygiene behaviour of households, the counterpart investments (in cash and/or kind) from households cannot be ignored either. Government staff from Community Water and Sanitation Agency (CWSA) and District Water and Sanitation Teams (DWST) may require at least US$ 1.8 per household/year for post intervention support services mainly monitoring, backstopping and sensitization for continuous improvement and sustainable positive change in hygiene behaviour.

It would be more insightful to compare reported findings here with the long-term effect or impact from the intervention to see any potential balance between cost and creditable achievements after a significant time of post HPI implementation.

## Declarations

### Author contribution statement

Paa Kwesi Woode, Bismark Dwumfour-Asare, Kwabena Nyarko: Conceived and designed the experiments; Performed the experiments; Analyzed and interpreted the data; Contributed reagents, materials, analysis tools or data; Wrote the paper.

Eugene Appiah-Effah: Performed the experiments; Analyzed and interpreted the data; Wrote the paper.

### Funding statement

This work was supported by the IRC/KNUST WASHCost Research Project through the Department of Civil Engineering of the Kwame Nkrumah University of Science and Technology.

### Competing interest statement

The authors declare no conflict of interest.

### Additional information

No additional information is available for this paper.
